# Dietary Supplementation with Algal-Derived Oligosaccharides: Impacts on the Growth Potential, Systemic Immunity, and Cecal Microbiota of Weaned Piglets

**DOI:** 10.3390/ani16101453

**Published:** 2026-05-09

**Authors:** Jianpu Zhao, Yiping Xie, Haichuan Lin, Lei Kong, Weiren Yang, Min Zhou, Shuzhen Jiang

**Affiliations:** 1 Key Laboratory of Efficient Utilization of Non-Grain Feed Resources (Co-Construction by Ministry and Province), Ministry of Agriculture and Rural Affairs, College of Animal Science and Technology, Shandong Agricultural University, Tai’an 271017, China; z1586208099@163.com (J.Z.); xyz246zzz@163.com (Y.X.); wryang@sdau.edu.cn (W.Y.); 2 Promotion Center for Animal Husbandry Development in Pingyi County, Linyi 273300, China; z64036209@163.com; 3 Shandong Provincial Animal Husbandry General Station, Shandong Provincial Quality Testing Station for Breeding Livestock and Poultry, Jinan 250109, China; kongleiisme@163.com

**Keywords:** piglet, oligosaccharide from algae, production performance, immune function, microorganisms

## Abstract

Sustainable piglet farming faces challenges from weaning stress, which necessitates the exploration of effective, natural antimicrobial alternatives to enhance growth and gut health. So, this study evaluated the effects of algal oligosaccharide (OSA) supplementation (0, 200, 400, 600, 800 mg/kg) as a natural antimicrobial alternative. Results showed that OSA significantly (*p* < 0.05) improved slaughter performance and the apparent digestibility of nutrients (OM, CP, amino acids) and energy. OSA increasing serum glucose while decreasing urea nitrogen and cholesterol, suggesting a shift in energy substrate mobilization. Enhanced antioxidant capacity was evidenced by increased T-SOD and GSH-Px activities and reduced MDA content. Immunologically, OSA elevated serum IgA, IgM, and IL-10, while inhibiting pro-inflammatory cytokines (IL-1β, IL-6). In the jejunum, OSA obviously increased disaccharidase activities and sIgA levels. Microbial analysis revealed that OSA favorably modulated cecal microbiota by increasing the abundance of Firmicutes and beneficial genera while decreasing Proteobacteria and potential pathogens (e.g., *Escherichia*). In conclusion, dietary OSA improved antioxidant capacity, and immunity while optimizing nutrient utilization and gut microbiota. The optimal dosage was determined to be 600–800 mg/kg. These findings suggest that OSA serves as a promising antimicrobial alternative in piglet farming.

## Simple Summary

This study addresses the challenges faced by young pigs during weaning, a stressful period that often leads to poor growth and health issues. To find natural alternatives to traditional antibiotics, we tested the effects of adding various amounts of seaweed-derived sugars, known as oligosaccharides, to the diets of 300 piglets. Our results showed that these seaweed sugars significantly improved the animals’ growth and their ability to digest essential nutrients and energy. The piglets showed stronger immune systems and better antioxidant protection, which helps fight off diseases and cell damage. Specifically, the seaweed sugars promoted a healthier gut by increasing beneficial bacteria and reducing harmful ones that cause infections. In the small intestine, the treatment also boosted enzymes that help break down food. In conclusion, adding 600 to 800 mg of seaweed sugars per kilogram of feed effectively enhances the health, immunity, and growth of piglets. These findings are valuable to society as they provide a sustainable and natural way to improve animal welfare and food production while reducing the reliance on antibiotics in farming.

## 1. Introduction

Weaning stress is a primary factor compromising piglet health from early development [1]. The abrupt dietary and environmental transitions associated with weaning adversely affect the gastrointestinal tract and immune system of piglets [2]. While, antibiotics have historically been employed to maintain health and enhance production performance [3], the accumulation of residual antibiotics contributes to the emergence of bacterial resistance [4]. This phenomenon poses significant risks to both animal welfare and human public health. With the prohibition of antibiotics, it is crucial to identify natural alternatives to antibiotics for improving piglets’ health.

An accumulating body of research indicates that oligosaccharide derived from algae (OSA) possess anti-tumor [5], antiviral, anti-bacterial, prebiotic and other biological activities [6]. Algae are classified into green, brown and red types based on their chemical composition [7], with OSA representing a major structural constituent of their cell walls [8]. Specifically, laminarin, alginates, and fucoidan are primarily derived from brown macroalgae, whereas ulvan and carrageenan are isolated from green and red algae, respectively, representing the major functional oligosaccharides in these marine plants [9]. A recent study indicated that OSA enhances the total antioxidant capacity in the serum of weaned piglets [10]. Furthermore, brown algae, rich in vitamins, minerals, and cellulose, have been proven to improve intestinal morphology and increase the diversity of beneficial cecal microbiota [11]. Additional study suggested that OSA functions as a prebiotic by inhibiting *Salmonella* colonization, thereby improving both intestinal health as well as production performance in broilers [12]. Despite these findings, limited research has comprehensively evaluated the influences of dietary OSA supplementation on immune function as well as cecal microorganisms of piglets.

We hypothesized that dietary OSA supplementation would improve nutrient digestibility and modulate cecal microbiota composition, thereby enhancing the immune status of weaned piglets without compromising growth performance. So, we aimed at investigate the influences of OSA on production performance, immune function, as well as cecal microorganisms in piglets. Our findings offer fresh perspectives on the utilization of OSA as a pioneering dietary supplement to bolster the physiological well-being of weaned piglets.

## 2. Materials and Methods

Algae oligosaccharides (Oligosaccharide from algae, OSA): Extracted from brown algae plants such as kelp, wakame, giant kelp, and Sargassum, and enzymatically prepared into brown algae oligosaccharides, with functional oligosaccharide content accounting for 66.41%, including: disaccharides 4.95%, trisaccharides 12.11%, tetrasaccharides 16.58%, pentasaccharides 13.54%, hexasaccharides 19.23%, provided by Wuzhou Feng Agricultural Technology Co., Ltd. in Yantai, China.

### 2.1. Animals, Diets, and Management

Three hundreds of Duroc × (Landrace × Yorkshire) piglets with an average initial weight of 11.84 ± 0.21 kg (weaned at 28 d) were enrolled in this 32-day study. After a seven-day adjustment period, animals were assigned to five dietary cohorts (*n* = 6 replicates per group, ten piglets per replicate): a control group receiving a basal diet (CON), other four groups provided with 200 (OSA200), 400 (OSA400), 600 (OSA600), or 800 (OSA800) mg/kg OSA. Six pigs were randomly selected from each group for sampling and indicator testing. The basal diet shown in Table 1 was formulated with reference to the National Research Council (NRC 2012, USA). Throughout the trial, piglets were housed in a controlled environment (30 °C initially, transitioning to 26–28 °C; ~65% humidity) with ad libitum access to water and feed. Standard vaccination and sanitation protocols were strictly implemented.

### 2.2. Sampling

Two piglets in each replicate were fasted for twelve hours before sampling. Blood was drawn from each pig via precaval vein puncture and transferred into 10 mL centrifuge tube. Following processing, the resulting serum was utilized to quantify systemic parameters, including oxidative stress markers, immunoglobulins, and pro-inflammatory/anti-inflammatory cytokines, alongside standard biochemical profiles. Mid-jejunum sample was used to test jejunal barrier index and disaccharidase activity. Subsequently, the liver, lungs, and spleen were promptly extracted and weighed, then calculated organ indexes. The organ index was expressed by organ weight/body weight (%). Additionally, the carcass weight was recorded to calculate the dressing percentage. Cecal contents were taken from each piglet for further analysis.

### 2.3. Determination of Growth Performance

Piglet performance was evaluated by weighing two representative animals per pen on days 8 and 49 to determine average daily gain (ADG). Weekly feed disappearance was recorded to calculate the average daily feed intake (ADFI). Efficiency of feed utilization was then expressed as the feed-to-gain ratio (F/G), calculated as the total feed intake divided by the total weight gain during the 41-day experimental phase.

### 2.4. Calculation of Apparent Digestibility of Nutrients

To evaluate nutrient utilization, one representative piglet from each replicate was housed in a specialized metabolic cage from day 20 to 26. Apparent digestibility was assessed using the total fecal collection technique, following established protocols [13]. Daily records of feed disappearance and total excreta were maintained. To prevent nitrogen loss, a 20% aliquot of the daily collected feces and urine was acidified with 10% sulfuric acid and homogenized for crude protein (CP) determination. After a 7-day period, pooled samples were analyzed for dry matter (DM), organic matter (OM), ether extract (EE), and gross energy (GE) according to AOAC (2012) standards. Furthermore, an amino acid analyzer (Hitachi 835-50) was utilized to quantify both essential and non-essential amino acids (EAA/NEAA). “The metabolic efficiency of dietary protein was further assessed by calculating Net Protein Utilization (NPU) and Biological Value (BV). These indices were determined based on nitrogen balance parameters, where NI represents nitrogen intake, while FN and UN denote nitrogen excreted in feces and urine, respectively:

NPU = [(NI − FN − UN)/NI] × 100

BV = [(NI − FN − UN)/(NI − FN)] × 100

### 2.5. Serum Biochemistry

Serum metabolic profiles, including lipid parameters (High density lipoprotein (HDL), low density lipoprotein (LDL), triglyceride (TG), and total cholesterol (TC)), glucose (GLU), as well as blood urea nitrogen (BUN), were quantified utilizing a COBUS MIRA Plus automatic biochemical analyzer (Roche Diagnostic System Inc., Indianapolis, IN, USA).

### 2.6. Serum Antioxidant-Related Parameters

Serum antioxidant-related parameters including malondialdehyde (MDA), glutathione peroxidase (GSH-Px), and total superoxide dismutase (T-SOD) were measured via commercial kits (Suzhou Keming Biotechnology Co., LTD, Suzhou, China).

### 2.7. Quantification of Serum Immunoglobulins

The concentrations of serum immunoglobulin A (IgA, IgG, and IgM) were determined using specialized ELISA kits (Jiangsu Meimian Industrial Co., Ltd., Yancheng, China).

### 2.8. Measurement of Serum Cytokines

Interleukin 1β (IL-1β), interleukin 6 (IL-6), interleukin 10 (IL-10), and tumor necrosis factor α (TNF-α) were quantified following the protocols provided by the manufacturer (Jiangsu Meimian Industrial Co., Ltd., Yancheng, China).

### 2.9. Jejunum Barrier Function Index

Mucosal integrity and immune markers, including intestinal trefoil factor (TFF3), histamine (HM), secretory immunoglobulin A (sIgA), and transforming growth factor α (TGF-α), were detected according to ELISA reagents (Jiangsu Meimian Industrial Co., Ltd., Yancheng, China).

### 2.10. Jejunum Disaccharidase

Lactase (LA), sucrase (SA), and maltase enzyme (MA) were assayed with commercial kits from Nanjing Jiancheng Institute (Nanjing, China).

### 2.11. Cecal Microbial Analysis

Based on the evaluated growth performance, nutrient utilization, dressing percentage, serum antioxidant enzymes, and immunoglobulin indexes, CON, OSA600, and OSA800 treatments were chosen for microbiota analysis. Genomic DNA was isolated from cecal digesta using the CTAB (Cetyl trimethylammonium bromide) protocol. For microbial identification, the V4 hypervariable region of the 16S rRNA gene was targeted for amplification using specific primers (515f/806f). Following rigorous purification and quantification, sequencing libraries were constructed utilizing the TruSeq^®^ DNA PCR-Free kit (Illumina, Inc., San Diego, CA, USA). High-throughput sequencing was executed on the Illumina NovaSeq system, producing 250 bp paired-end reads. Sequence clustering into OTUs (97% identity) was performed via Uparse (v7.0.1001), while diversity indices and downstream visualizations were generated through QIIME (v1.9.1) and R (v2.15.3), respectively.

### 2.12. Statistical Analyses

Statistical evaluations of the collected data were executed using SAS 9.4 software (Institute Inc., Cary, NC, USA) via one-way analysis of variance (ANOVA). To explore the dose–response relationship of OSA, orthogonal polynomial contrasts were utilized to determine both linear and quadratic effects across all measured parameters. Post hoc comparisons among groups were performed using Duncan’s multiple-range test. Significant disparities were acknowledged at a probability level of *p* < 0.05, and all findings are presented as means and their associated standard error (SEM).

## 3. Results

### 3.1. Observed Growth Performance

Influences of OSA on growth performance in piglets are exhibited on Table 2. With the increasing of OSA (200, 400, 600 and 800 mg/kg), ADFI increased linearly (*p* < 0.05). However, no obvious effects of OSA on ADG and F/G ratio of piglets were observed (*p* > 0.05).

### 3.2. Apparent Digestibility Values

Dietary supplementation of OSA (200, 400, 600 and 800 mg/kg) exerted significant linear and quadratic enhancements on the apparent digestibility of OM, CP, and GE, as well as the nitrogen utilization efficiency (BV and NPU) (*p* < 0.05, Table 3). Conversely, DM digestibility exhibited a strictly linear upward trend (*p* < 0.05). Apparent digestibility of OM and CP, and apparent availability of CP including BV and NPU in the OSA600 and OSA800 was obviously higher than that in control (*p* < 0.05). Only the apparent digestibility of GE in OSA800 was obviously higher than that in control (*p* < 0.05). NPU in both OSA200 and OSA400 was obviously higher than that in control (*p* < 0.05).

### 3.3. Apparent Digestibility of EAA

Regarding amino acid utilization, the apparent digestibility of lysine (Lys), threonine (Thr), valine (Val) and total essential amino acids (TEAA) exhibited both linear and quadratic improvements as OSA levels rose (*p* < 0.05, Table 4). While apparent digestibility of leucine (Leu) and isoleucine (Ile) responded to OSA supplementation in a strictly linear manner (*p* < 0.05). Compared with control group, piglets in the OSA600 group showed markedly enhanced digestibility across all these amino acids (Lys, Leu, Ile, Thr, Val and TEAA) (*p* < 0.05). Furthermore, the OSA800 treatment also maintained significantly superior digestibility for Lys, Leu, Ile as well as TEAA relative to the control group (*p* < 0.05).

### 3.4. Apparent Digestibility of NEAA

With increasing OSA addition, significant linear and quadratic increments were observed in the apparent digestibility of aspartic acid (Asp), serine (Ser), proline (Pro), cystine (Cys) and total NEAA increased (*p* < 0.05, Table 5). Conversely, the digestibility of glycine (Gly) and tyrosine (Tyr) showed a strictly linear upward trend (*p* < 0.05). The apparent digestibility of Asp, Ser, Pro, Cys and total NEAA in the OSA600 and OSA800 was obviously higher than that in control (*p* < 0.05). It is also worth noting that the OSA400 dose specifically enhanced Cys digestibility relative to the CON group (*p* < 0.05).

### 3.5. Slaughter Performance

With the increasing of inclusion of dietary OSA, the dressing percentage increased linearly and quadratically (*p* < 0.05, Table 6). Dressing percentage was markedly improved in both OSA600 and OSA800 groups relative to control group (*p* < 0.05). In contrast, dietary OSA inclusion failed to alter the visceral development of piglets, as evidenced by the statistically similar indices for the liver, lung, spleen, and kidney across all experimental groups (*p* > 0.05).

### 3.6. Serum Biochemical Index Values

With OSA addition increasing in diet, the content of HDL and TG increased linearly and quadratically (*p* < 0.05, Table 7), and GLU content only increased quadratically (*p* < 0.05), while content of TC and BUN reduced linearly and quadratically (*p* < 0.05). The TG in both OSA600 and OSA800, and the GLU in the OSA200, OSA400 and OSA600 were obviously higher than those in control (*p* < 0.05). The TC in the OSA600 and OSA800, and the BUN in the OSA400, OSA600 and OSA800 were markedly reduced compared to control (*p* < 0.05).

### 3.7. Serum Antioxidant Enzyme

With increasing dietary inclusion of OSA, activity of T-SOD and GSH-Px increased linearly and quadratically (*p* < 0.05, Table 8), but MDA content reduced linearly and quadratically (*p* < 0.05). Specifically, OSA800 exhibited markedly superior T-SOD activity compared to OSA200, OSA400 and control (*p* < 0.05). GSH-Px levels across all OSA-supplemented groups consistently surpassed those of the control (*p* < 0.05). MDA accumulation in the high-dose OSA treatments (600 and 800 mg/kg) was significantly lower than in the lower-dose and control groups (*p* < 0.05).

### 3.8. Serum Immunoglobulins Levels

As dietary OSA addition increased, serum IgA as well as IgM increased linearly and quadratically (*p* < 0.05, Table 9). Significantly higher IgA level was observed in the OSA600 and OSA800 groups compared to both OSA200 and OSA400 groups, while the latter two groups also showed elevated IgA relative to the control (*p* < 0.05). For IgM, level in OSA600 and OSA800 treatments was obviously higher than that in OSA200 and control (*p* < 0.05).

### 3.9. Serum Cytokines

With inclusion of OSA in diet increasing, the serum IL-1β decreased linearly and quadratically (*p* < 0.05, Table 10), while IL-6 levels decreased quadratically. Conversely, a quadratic enhancement in IL-10 was observed (*p* < 0.05). The IL-1β in all experimental treatments, and the IL-6 in the OSA400, OSA600 and OSA800 were obviously lower than that in control (*p* < 0.05). Regarding the anti-inflammatory mediator IL-10, levels in the OSA600 and OSA800 cohorts significantly surpassed those in the lower-dose groups (200 and 400 mg/kg), which in turn were consistently higher than the control (*p* < 0.05).

### 3.10. Jejunal Barrier Function Indices

With the increase in OSA dosage, jejunal TFF3 concentrations exhibited significant linear and quadratic reductions, whereas sIgA and TGF-α levels displayed significant linear and quadratic increase (*p* < 0.05, Table 11). Notably, TFF3 of OSA600 and OSA800 groups was significantly lower than that of OSA400 (*p* < 0.05), which in turn was lower than the OSA200 group, and ultimately the control (*p* < 0.05). The changing trends of sIgA and TFF3 are completely opposite. The TGF-α levels in OSA600 and OSA800 groups were significantly higher than those in OSA200 and OSA400 (*p* < 0.05), and in the OSA200 and OSA400 was obviously higher than that in control (*p* < 0.05).

### 3.11. Jejunal Disaccharidase Activity

With increasing OSA addition, the activity of lactase, sucrase and maltase showed a clear dose-dependent increment (*p* < 0.05, Table 12). Lactase and sucrase in the OSA600 and OSA800 surpassed those in OSA400 (*p* < 0.05), and a prominent upregulation was observed in OSA400 relative to OSA200 and control (*p* < 0.05). The maltase in OSA600 and OSA 800 was obviously higher than that in OSA200 and control (*p* < 0.05).

### 3.12. Cecal Microbial Diversity

**Figure 1:**
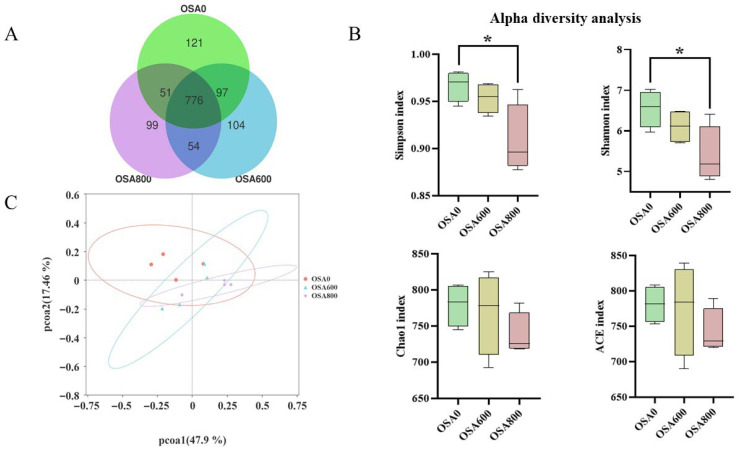
Venn diagram of OTUs (**A**); Comparison of alpha diversity analysis in different treatments (**B**); PCoA shows difference in microbial community structure in different treatments (**C**). Data are mean ± standard error of the mean (*n* = 4). Significant differences are displayed in the figures by * *p* < 0.05.

As shown in Venn diagram (Figure 1A), the control and OSA600 shared the most OTUs, which was 97, while the control and OSA800 shared the least OTUs, which was 51. The total OTUs shared by the three treatments was 776. Compared with control (Figure 1B), the Shannon index and Simpson index of OSA800 treatment were significantly decreased (*p* < 0.05), while the Chao1 index and ACE index had no obvious difference (*p* > 0.05). No significant change in the Simpson, Shannon, Chao1 and ACE indexes between control and OSA600 treatment (*p* > 0.05). The PCoA analysis (Figure 1C) revealed that OSA600 values dispersed relatively far apart from the control and OSA800, suggesting the difference in microbial composition at different OSA levels.

### 3.13. Relative Abundance of Cecal Microorganisms

**Figure 2:**
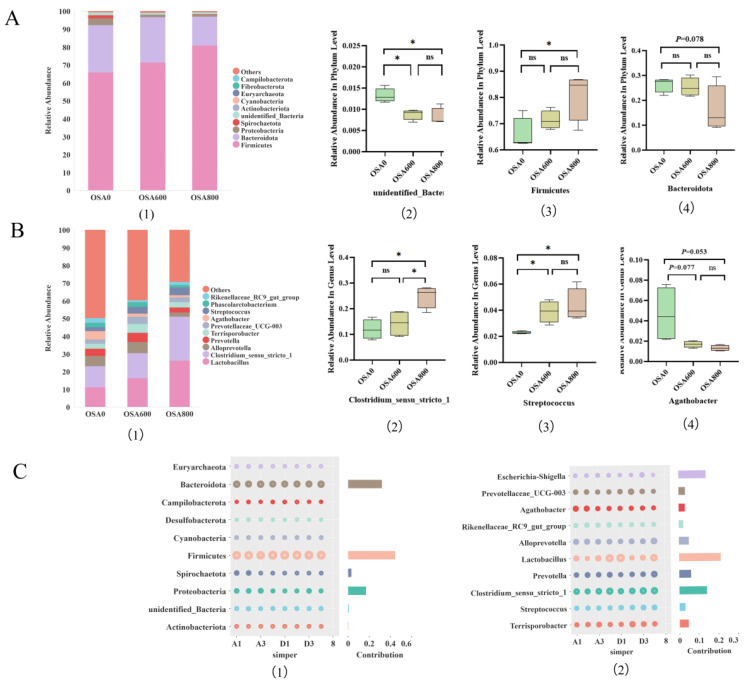
The Relative abundance (%) of dominant species of microorganisms and differential bacteria at phylum level (**A**(**1**–**4**)). The relative abundance (%) of dominant species of microorganisms and differential bacteria at genus level (**B**(**1**–**4**)). Simper difference contribution analysis chart of horizontal species of phylum and genus (**C**(**1**,**2**)). Data are mean ± standard error of the mean (*n* = 4). Significant differences are displayed in the figures by * *p* < 0.05.

Relative abundance (top 10) of the cecal microbiota at the phylum level, along with the top three cecal microorganisms were shown in Figure 2A. The *Firmicutes* and *Bacteroidota* were the most abundant at the phylum level. Compared to OSA0, abundance of *Firmicutes* increased significantly in OSA800 (*p* < 0.05), whereas the abundance of *Bacteroidota* showed a downward trend in OSA800 (*p* = 0.078). And the abundance of *unidentified_Bacteria* significantly decreased in OSA600 and OSA800 (*p* < 0.05). Relative abundance (top 10) of cecal microbiota at the genus level and top three cecal microorganisms were shown in Figure 2B. The *Lactobacillus* and *Clostridium_sensu_stricto_1* were the most abundant at the genus level. Compared to OSA0, the abundance of Streptococcus increased significantly in OSA600 and OSA800 (*p* < 0.05), while abundance of *Agathobacter* had a downward trend in OSA600 (*p* = 0.077) and OSA800 (*p* = 0.053) groups. And abundance of *Clostridium_sensu_stricto_1* increased obviously in OSA800 compared with OSA0 and OSA600 (*p* < 0.05). The simper difference contribution analysis was shown in Figure 2C. At the genus level, the simper difference was more contributed by *Lactobacillus*, *Clostridium_sensu_stricto_1*, *Escherichia-Shigella*, *Prevotellaceae_UCG-003*, *Alloprevotella* and *Terrisporobacter*.

### 3.14. Correlation Analysis

**Figure 3:**
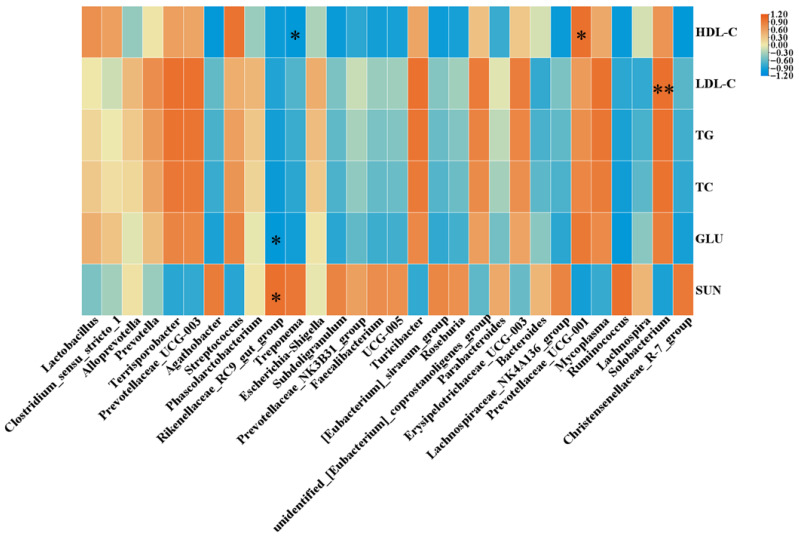
Heat map of correlation analysis between cecal microflora at genus level and serum biochemical indices (*n* = 4). Significant differences are displayed in the figures by * *p* < 0.05 and ** *p* < 0.01.

As shown in Figure 3, the *Prevotellaceae_UCG-001* showed a significant positive correlation with HDL (*p* < 0.05), while the *Treponema* exhibited a significant negative correlation with HDL (*p* < 0.05). The *Solobacterium* showed a highly significant positive correlation with LDL (*p* < 0.01). The *Rikenellaceae_RC9_gut* group had a significant negative correlation with GLU (*p* < 0.05), but a positive correlation with BUN (*p* < 0.05).

## 4. Discussion

### 4.1. Growth Performance

OSA are alginate-derived degradation products characterized by low molecular weight and high water solubility, exhibiting significant anti-inflammatory and antioxidant properties [11]. Effects of OSA on animal growth performance appear to vary depending on the source and dosage. Some studies reporting increased ADG and ADFI [13] and others showing no significant effect [12], which experiment found that OSA did not markedly improve growth performance.

In terms of nutrient utilization, previous studies have demonstrated that OSA enhance nutrient digestibility. Wan et al. (2017) found that OSA can also improve apparent digestibility of CP, CA and CF [13]. Similarly, Nochta et al. (2010) found that dietary mannan-oligosaccharide significantly improved the absorption of certain amino acids namely lysine, methionine, cystine, and threonine [14]. Consistent with these reports, higher OSA does (600, 800 mg/kg) were associated with significantly improved apparent digestibility of OM and CP compared to CON [15]. Furthermore, 600 and 800 mg/kg supplementation enhanced the apparent digestibility of specific EAA (Lys, Leu and Ile), as well as NEAA (Asp, Ser, Pro and Cys). These results collectively indicate that high-does OSA positively influences nutrient digestibility in piglets, though its underlying mechanisms warrant further investigation.

### 4.2. Oxidative Stress

MDA serves as a key indicator of lipid peroxidation, while T-SOD and GSH-Px are essential for neutralizing free radicals [16,17,18]. Previous studies on oligosaccharides (OSA/IMO) have yielded inconsistent results regarding their effects on SOD and MDA in piglets [13,19]. In the present study, high-dose OSA (800 mg/kg) significantly enhanced T-SOD activity, while concentrations of 600–800 mg/kg effectively reduced MDA levels. Furthermore, GSH-Px activity was upregulated across all OSA inclusion levels (200–800 mg/kg).

Considering that the Nrf2 signaling pathway, often activated via the p62/SQSTM1 non-canonical route, is a master regulator of ROS homeostasis [20], whether OSA utilizes this pathway to mitigate oxidative stress in piglets remains to be determined and requires further experimental validation. Overall, dietary OSA supplementation improves the antioxidant status of weaned piglets by activating key antioxidant enzymes and eliminating peroxidative metabolites. The precise molecular mechanisms governing these effects warrant further investigation through integrated in vivo and in vitro studies.

### 4.3. Serum Biochemical Indices

Serum biochemical markers are essential indicators of lipid, glucose, and protein metabolism. HDL facilitates reverse cholesterol transport, while TG and TC reflect lipid storage and systemic lipid proficiency, respectively [21,22,23,24,25]. GLU levels indicate glucose homeostasis, and BUN serves as a key marker for protein catabolism and nitrogen utilization [26,27].

Recent studies on oligosaccharides have reported varied metabolic responses [19,28,29]. In the present study, dietary OSA supplementation led to a linear and quadratic increase in HDL levels, suggesting that OSA may promote cholesterol metabolism via enhanced reverse transport. While TG levels increased significantly, they remained within the physiological range (20–21 mg/dL), far below the hypertriglyceridemia threshold (>150 mg/dL) [30]. Furthermore, high-dose OSA (600 and 800 mg/kg) significantly reduced TC levels, aligning with previous findings in poultry and swine [31,32,33]. Regarding glucose metabolism, we observed significant increases in GLU levels across all OSA treatments. This trend is consistent with effects observed with chitosan oligosaccharides [34,35,36] and suggests an enhancement in systemic glucose mobilization or metabolic efficiency. In terms of protein metabolism, OSA supplementation led to a decrease in BUN levels, consistent with the findings of Tang et al. [37]. This reduction indicates improved nitrogen utilization and enhanced amino acid incorporation into protein synthesis, reflecting more efficient protein metabolism in OSA-supplemented piglets.

In conclusion, dietary OSA positively modulates lipid and protein metabolism and enhances glucose availability in weaned piglets, though the specific regulatory pathways require further investigation.

### 4.4. Immune Function

Immunoglobulins are fundamental to immune defense: IgA provides critical protection at mucosal surfaces, IgG maintains systemic immune stability, and IgM functions as a potent multimeric defense mechanism [38,39,40,41,42]. Consequently, fluctuations in serum immunoglobulin concentrations often serve as indicators of overall immune status. Previous studies have shown that 100 mg/kg oligochitosan improves serum IgG, IgA, and IgM levels in broilers [43], while chitosan oligosaccharide supplementation (100–600 mg/kg) in lambs specifically elevates IgM concentrations [44].

In the present study, OSA supplementation at 200–800 mg/kg significantly increased serum IgA levels, while IgM concentrations were notably elevated in the 400–800 mg/kg treatment groups. These findings suggest that dietary OSA at concentrations of 400 mg/kg or higher enhances the immune response in weaned piglets. Given that oligosaccharide-induced IgM can bind to bacteria and stimulate innate immune cell activation [45], it is hypothesized that OSA augments host immunity by enhancing the viability and functional activity of immune cells.

### 4.5. Inflammatory Factors

Interleukins exert a crucial role in immune regulation various biology processes, with fluctuations in their levels often serving as indicators of disease states. As a key anti-inflammatory mediator, IL-10 is essential for maintaining immune homeostasis and mitigating inflammatory damage. Previous research has demonstrated that κ/β-carrageenan-derived oligosaccharides from red algae can upregulate circulating IL-10 concentrations in mice [46]. Consistent with these findings, the present study demonstrated that high concentrations of OSA significantly increased IL-10 level. IL-6 is a pleiotropic cytokine typically released into the circulation in response to tissue inflammation or homeostasis disruption [47]. In this study, OSA reduced serum IL-6 in piglets, indicating its potential to alleviate inflammation and subsequently enhance growth performance. Similarly, both TNF-α and IL-1β are potent pro-inflammatory cytokines that trigger the production of multiple inflammatory mediators and provoke multiple inflammatory cascades [48,49]. This study observed that serum TNF-α and IL-1β in piglets decreased progressively as the dietary OSA level increased.

These results collectively indicate that OSA alleviates inflammatory responses, contributing to improved vitality in piglets. Supporting this, other studies have reported that alginate oligosaccharide reduces TNF-α levels and its mRNA expression in jejunum of pigs [50,51]. Such anti-inflammation effects likely contribute to the maintenance of intestinal barrier integrity in pigs.

### 4.6. Jejunal Indices

Beyond its anti-inflammatory effects, OSA was found to enhance intestinal barrier integrity and digestion function. Indicators of mucosal repair suggested a reduction in intestinal damage, evidenced by decreased TFF3 level (a marker typically upregulated following injury) and an increased level of the healing-promoting factor TGF-α in the jejunum [52,53]. Furthermore, intestinal immune defense was strengthened, as high-dose OSA markedly elevated jejunal sIgA content. This observation is consistent with existing literature [54], identifies sIgA as a key immunoglobulin that protects the mucosal surface from pathogens and serves as a critical indicator of gut immune competence [55].

Finally, nutrient digestion was significantly improved. We observed a dose-responsive increase in the activities of lactase, sucrase, and maltase in the jejunum. These findings, which confirms earlier reports [54,55,56], demonstrate that OSA enhances carbohydrate digestion and absorption capacity in weaned piglets, likely by fostering a healthier intestinal milieu.

### 4.7. Cecal Microbiota

The intestinal microbiome is essential for maintaining animal health and changes in diet and environment post-birth can significantly alter the microbial landscape in piglets [57]. During the transition from lactation to weaning, the gut microbial community undergoes a characteristic shift, the abundance of exogenous pathogens like Escherichia coli, Salmonella and streptococcus increases, while beneficial endogenous lactic acid bacteria and anaerobic bacteria decrease [58]. Such microbial imbalance is a primary contributor to post-weaning diarrhea [59]. OSA supplementation reversed this trend by significantly increasing abundance of health-promoting Firmicutes, containing *Lactobacillus*, *Clostridium_sensu_stricto_1* and *Streptococcus* increased, while suppressing potential pathogens like *Escherichia-Shigella*, *Prevotella* and *Alloprevotella*. The abundance of specific taxa, such as *Lactobacillus*, was increased, while the implications of shifts in the *Streptococcus* genus are interpreted with caution due to its functional diversity. These findings align with reports indicating that diarrheal piglets exhibit reduced levels of Firmicutes and its beneficial genera [59]. The increase in *Lactobacillus*, a genus known to be stimulated by seaweed polysaccharides, is particularly noteworthy [60]. *Lactobacillus* inhibits the fermentation of undigested protein in the gut, thereby optimizing the intestinal environment and promoting the growth of other beneficial microbes [61]. Furthermore, beneficial bacteria like *Lactobacillus* produce polysaccharidases and glycosidases that degrade structural polysaccharides in plant cell walls, directly enhancing nutrient digestibility [62].

Therefore, the improved nutrient digestibility observed in OSA-supplemented pigs may be attributed to this induced microbial shift, characterized by a reduction in opportunistic pathogens and a proliferation of beneficial taxa. Collectively, above results indicate that OSA functions as an effective prebiotic to improve the intestinal environment of piglets. However, the present study is limited by the lack of long-term observation, and the underlying molecular mechanisms remain to be fully elucidated. Building upon these preliminary findings, further in-depth investigations are warranted to bridge these knowledge gaps.

## 5. Conclusions

In summary, this study confirms that OSA is a promising feed additive for enhancing piglet welfare and production efficiency. Its primary benefits involve the modulation of nutrient digestibility, antioxidant status, and immune-related parameters. The dose-responsive nature of these improvements including improved dressing percentage, reduced MDA, elevated IgA and IL-10, and increased beneficial cecal bacteria indicates that the optimal inclusion level for maximizing benefits under our experimental conditions is 600–800 mg/kg. Consequently, our results provide compelling evidence for the adoption of OSA as a novel nutritional intervention to ensure health and robustness of weaned piglets.

However, it is important to note that dietary OSA did not significantly affect growth performance (ADG and F/G) during the study period. While the changes in metabolic and microbial indicators are promising, they should be interpreted with caution as the underlying molecular mechanisms and long-term effects were not fully elucidated. Consequently, our results suggest that OSA is a beneficial nutritional intervention for bolstering the health and robustness of weaned piglets, although further research is warranted to clarify its precise mode of action and confirm its efficacy in large-scale production settings.

## Figures and Tables

**Table 1:** Ingredients and nutrient contents of the basal diet (air-dry basis)%.

Ingredients	Content	Nutrients ^2^	
Expanded corn	68.69	DE MJ/kg	14.02
Soybean meal CP 46%	13.30	ME MJ/kg	13.46
Fermented soybean meal	6.30	Crude protein	18.01
Expanded soybean	7.00	Calcium	0.82
Plasma protein	0.50	Total phosphorus	0.66
CaHPO_4_	0.86	Available phosphorus	0.44
Pulverized Limestone	1.07	Lys	1.44
NaCl	0.45	Met	0.44
L-Lysine HCl	0.55	Sulfur amino acid	0.85
DL-Met	0.16	Thr	0.87
L-Thr	0.12	Trp	0.22
Premix ^1^	1.0		
Total	100		

^1^ Supplied per kg of diet: VA 2 300 IU; VD3 230 IU; VE 20 IU; VK3 0.60 mg; VB1 1.80 mg; VB2 4.25 mg; Pantothenic acid 13.00 mg; Nicotinic acid 20.00 mg; Pyridoxine 2.00 mg; Biotin 0.09 mg; Folic acid 0.45 mg; VB12 0.02 mg; Mn (Mn-Met) 6.00 mg; Fe (Iron (II) fumarate) 150 mg; Zn (Zn-Gly) 150 mg; Cu (Cu-Gly) 9.00 mg; I (Calcium iodate) 0.21 mg; Se (Selenium-Rich Yeas) 0.45 mg. ^2^ Digestible energy, crude protein, calcium and available phosphorus were analyzed values, while the other nutrient levels were calculated values.

**Table 2:** Effects of oligosaccharide from algae (OSA) on the growth performance of piglets (*n* = 6).

Items		ADFI g/d	ADG g/d	F/G
CON		919.7	436.2	2.113
OSA200		926.6	455.3	2.04
OSA400		945.5	462.8	2.051
OSA600		938.6	457.9	2.055
OSA800		948.8	458.9	2.076
SEM		4.909	4.984	0.025
*p*-Value	Treatment	0.307	0.492	0.893
	Linear	0.045	0.174	0.729
	Quadratic	0.125	0.198	0.632

CON was a basal diet, OSA200, OSA400, OSA600, and OSA800 were basal diet supplemented with 200, 400, 600, and 800 mg/kg OSA, respectively. The same below. ADFI, Average daily feed intake; ADG, Average daily Gain; F/G, ADFI (g)/ADG (g). SEM, Standard error of the means.

**Table 3:** Effects of oligosaccharide from algae (OSA) on the apparent digestibility and availability of nutrients (*n* = 6) %.

Items		DM	OM	CP	EE	GE	BV	NPU
CON		87.35	87.20 ^b^	82.48 ^b^	92.51	87.24 ^b^	67.60 ^b^	55.76 ^c^
OSA200		87.86	87.37 ^b^	83.86 ^b^	92.64	87.75 ^ab^	68.17 ^ab^	57.17 ^b^
OSA400		87.76	87.72 ^ab^	84.09 ^ab^	92.42	87.80 ^ab^	69.68 ^ab^	58.59 ^ab^
OSA600		87.54	88.06 ^a^	84.48 ^a^	92.03	87.66 ^ab^	70.17 ^a^	59.27 ^a^
OSA800		88.82	88.87 ^a^	84.73 ^a^	92.95	88.87 ^a^	70.48 ^a^	59.71 ^a^
SEM		0.153	0.133	0.202	0.136	0.14	0.106	0.143
*p*-Value	Treatment	0.053	0.004	0.017	0.325	0.016	<0.001	<0.001
	Linear	0.023	0.019	0.001	0.797	0.004	<0.001	<0.001
	Quadratic	0.054	0.042	0.003	0.476	0.011	<0.001	<0.001

DM, Dry matter; OM, Organic matter; CP, Crude protein; EE, Ether extract; GE, Gross energy; BV, Biological value; NPU, Net protein utilization. SEM, Standard error of the means. ^a,b,c^ Different letters mean significantly different (*p* < 0.05).

**Table 4:** Effects of oligosaccharide from algae (OSA) on the apparent digestibility of essential amino acids (*n* = 6) %.

Items		Lys	Met	Arg	His	Leu	Ile	Phe	Thr	Val	TEAA
CON		83.38 ^b^	87.84	91.78	87.19	84.71 ^b^	80.20 ^b^	84.43	82.33 ^b^	81.39 ^b^	84.61 ^b^
OSA200		84.28 ^ab^	88.55	91.67	87.18	84.97 ^b^	80.57 ^b^	84.43	82.73 ^b^	81.80 ^b^	84.92 ^ab^
OSA400		84.09 ^ab^	88.47	91.61	87.02	85.00 ^b^	80.67 ^b^	84.66	83.10 ^ab^	82.13 ^b^	85.11 ^ab^
OSA600		85.42 ^a^	89.65 ^a^	92.72	87.73	86.64 ^a^	82.09 ^a^	85.73	84.13 ^a^	84.33 ^a^	86.15 ^a^
OSA800		84.93 ^a^	88.99 ^a^	92.21	88.23	85.85 ^a^	81.71 ^a^	85.5	83.81 ^ab^	82.93 ^ab^	85.89 ^a^
SEM		0.164	0.352	0.184	0.211	0.174	0.218	0.169	0.248	0.164	0.352
*p*-Value	Treatment	0.006	0.583	0.201	0.297	0.011	0.049	0.238	0.012	0.008	0.024
	Linear	0.001	0.169	0.161	0.601	0.021	0.033	0.036	0.003	0.011	0.004
	Quadratic	0.005	0.346	0.361	0.102	0.065	0.073	0.114	0.013	0.033	0.016

Lys, Lysine; Met, Methionine; Arg, Arginine; His, histidine; Leu, leucine; Ile, isoleucine; Phe, Phenylalanine; Thr, threonine; Val, valine; TEAA, Total essential amino acids. SEM, Standard error of the means. ^a,b^ Different letters mean significantly different (*p* < 0.05).

**Table 5:** Effects of oligosaccharide from algae (OSA) on the apparent digestibility of non-essential amino acids (*n* = 6) %.

Items		Asp	Ser	Glu	Pro	Gly	Ala	Cys	Tyr	TNEAA
CON		84.58 ^b^	87.71 ^b^	91.11	84.94 ^b^	79.72	80.57	88.01 ^b^	83.05	85.13 ^b^
OSA200		84.89 ^ab^	88.00 ^ab^	91.03	85.02 ^b^	80.19	81.85	88.65 ^ab^	84.1	85.39 ^ab^
OSA400		85.16 ^ab^	88.40 ^ab^	91.76	86.10 ^ab^	79.93	81.15	89.75 ^a^	83.93	85.51 ^ab^
OSA600		86.74 ^a^	90.26 ^a^	92.08	87.81 ^a^	81.89	82.86	90.96 ^a^	84.16	87.35 ^a^
OSA800		85.81 ^a^	88.85 ^a^	91.61	86.04 ^a^	82.86	82.46	90.31 ^a^	84.78	86.33 ^a^
SEM		0.207	0.191	0.737	0.216	0.281	0.271	0.206	0.23	0.24
*p*-Value	Treatment	0.023	0.003	0.981	0.021	0.094	0.078	0.041	0.243	0.045
	Linear	0.008	0.045	0.683	0.023	0.037	0.019	0.015	0.033	0.037
	Quadratic	0.027	0.027	0.913	0.039	0.108	0.061	0.031	0.105	0.016

Asp, Aspartic acid; Ser, serine; Glu, glutamic acid; Pro, proline; Gly, glycine; Ala, alanine; Cys, Cystine; Tyr, Tyrosine; TNEAA, Total non-essential amino acids. SEM, Standard error of the means. ^a,b^ Different letters mean significantly different (*p* < 0.05).

**Table 6:** Effects of oligosaccharide from algae (OSA) on the slaughter rate and organ index of piglets (*n* = 6) %.

Items		Dressing Percentage	Liver Index	Renal Index	Lungndex	Spleen Index
CON		66.44 ^b^	2.67	0.46	1.28	0.183
OSA200		66.79 ^ab^	2.67	0.48	1.31	0.194
OSA400		67.02 ^ab^	2.69	0.49	1.32	0.192
OSA600		67.73 ^a^	2.67	0.48	1.31	0.194
OSA800		67.82 ^a^	2.68	0.47	1.31	0.194
SEM		0.945	0.531	0.006	0.053	0.051
*p*-Value	Treatment	0.016	0.999	0.924	1.002	1.011
	Linear	0.016	0.883	0.665	0.955	0.952
	Quadratic	0.034	0.989	0.832	0.997	0.997

SEM, Standard error of the means. ^a,b^ Different letters mean significantly different (*p* < 0.05).

**Table 7:** Effects of oligosaccharide from algae (OSA) on the serum biochemical index of piglets (*n* = 6).

Items		HDL mmol/L	LDL mmol/L	TG mg/mL	TC μmoL/dL	GLU μmol/mL	BUN mg/mL
CON		1.57	1.66	0.17 ^b^	34.36 ^a^	6.42 ^b^	0.31 ^a^
OSA200		1.56	1.64	0.17 ^b^	34.40 ^a^	6.94 ^a^	0.29 ^ab^
OSA400		1.63	1.64	0.18 ^ab^	32.51 ^a^	6.98 ^a^	0.27 ^bc^
OSA600		1.64	1.62	0.21 ^a^	27.83 ^b^	6.90 ^a^	0.25 ^c^
OSA800		1.64	1.63	0.20 ^a^	28.83 ^b^	6.73 ^ab^	0.25 ^c^
SEM		0.008	0.008	0.003	0.362	0.061	0.004
*p*-Value	Treatment	0.127	0.609	<0.001	<0.001	0.046	<0.001
	Linear	0.021	0.408	<0.001	<0.001	0.24	<0.001
	Quadratic	0.027	0.393	<0.001	<0.001	0.01	<0.001

HDL, High density lipoprotein; LDL, Low density lipoprotein; TG, Triacylglyceride; TC, Total cholesterol; GLU, Glucose; BUN, Serum urea nitrogen. SEM, Standard error of the means. ^a,b,c^ Different letters mean significantly different (*p* < 0.05).

**Table 8:** Effects of oligosaccharide from algae (OSA) on the serum antioxidant enzymes of piglets (*n* = 6).

Items		T-SOD U/mL	GSH-Px U/mL	MDA nmol/mL
CON		90.70 ^b^	107.60 ^b^	0.58 ^a^
OSA200		92.40 ^b^	119.80 ^a^	0.58 ^a^
OSA400		93.80 ^b^	120.60 ^a^	0.57 ^a^
OSA600		96.52 ^ab^	123.00 ^a^	0.50 ^b^
OSA800		104.21 ^a^	129.00 ^a^	0.48 ^b^
SEM		1.241	1.546	0.006
*p*-Value	Treatment	0.018	0.004	0.019
	Linear	0.001	<0.001	0.008
	Quadratic	0.002	0.001	0.015

T-SOD, Total superoxide dismutase; GSH-Px, Glutathione peroxidase; MDA, Malondialdehyde. SEM, Standard error of the means. ^a,b^ Different letters mean significantly different (*p* < 0.05).

**Table 9:** Effects of oligosaccharide from algae (OSA) on the serum immune globulin of piglets (*n* = 6) μg/mL.

Items		IgA	IgG	IgM
CON		34.18 ^c^	362.99	38.27 ^b^
OSA200		39.03 ^b^	363.75	38.40 ^b^
OSA400		40.85 ^b^	366.78	40.21 ^ab^
OSA600		47.03 ^a^	376.21	41.74 ^a^
OSA800		46.28 ^a^	375.76	41.61 ^a^
SEM		1.033	2.762	0.421
*p*-Value	Treatment	<0.001	0.389	0.002
	Linear	<0.001	0.07	<0.001
	Quadratic	<0.001	0.18	<0.001

IgA, Immunoglobulin A; IgG, Immunoglobulin G; IgM, Immunoglobulin M. SEM, Standard error of the means. ^a,b,c^ Different letters mean significantly different (*p* < 0.05).

**Table 10:** Effects of oligosaccharide from algae (OSA) on the serum cytokines of piglets (*n* = 6).

Items		IL-1β ng/L	IL-6 ng/L	IL-10 ng/L	TNF-α pg/mL
CON		45.94 ^a^	1394.38 ^a^	159.62 ^c^	424.64
OSA200		43.56 ^b^	1334.97 ^a^	176.19 ^b^	422.41
OSA400		43.38 ^b^	1263.48 ^b^	179.93 ^b^	421.32
OSA600		42.81 ^b^	1236.93 ^b^	200.24 ^a^	415.64
OSA800		41.86 ^b^	1199.40 ^b^	191.36 ^a^	413.72
SEM		0.305	10.337	1.672	1.583
*p*-Value	Treatment	0.005	<0.001	<0.001	0.176
	Linear	<0.001	0.634	<0.001	0.012
	Quadratic	0.001	0.001	<0.001	0.043

IL-1β, Interleukin-1β; IL-6, Interleukin-6; IL-10, Interleukin-10; TNF-α, Tumor necrosis factor alpha. SEM, Standard error of the means. ^a,b,c^ different letters mean significantly different (*p* < 0.05).

**Table 11:** Effects of oligosaccharide from algae (OSA) on the jejunal barrier function of piglets (*n* = 6).

Items		TFF3 ng/mL	HM U/L	sIgA ug/mL	TGF-α pg/mL
CON		2246.50 ^a^	2053.07	1848.51 ^d^	1900.63 ^c^
OSA200		2133.09 ^b^	1892.95	1941.27 ^c^	1988.66 ^b^
OSA400		2049.56 ^c^	1854.34	2103.38 ^b^	2052.91 ^b^
OSA600		1922.07 ^d^	1647.29	2295.91 ^a^	2212.48 ^a^
OSA800		1877.90 ^d^	1771.17	2327.51 ^a^	2259.17 ^a^
SEM		5.875	31.158	7.801	6.905
*p*-Value	Treatment	<0.001	0.128	<0.001	<0.001
	Linear	0.002	0.02	0.005	<0.001
	Quadratic	0.034	0.042	0.013	<0.001

TFF3, Trefoil factor family 3; sIgA, Secreted immunoglobulin A; HM, histamine; TGF-α, Transforming growth factor alpha. SEM, Standard error of the means. CON was a basal diet, OSA200, OSA400, OSA600, and OSA800 were basal diet supplemented with 200, 400, 600, and 800 mg/kg OSA, respectively. ^a,b,c,d^ Different letters mean significantly different (*p* < 0.05).

**Table 12:** Effects of oligosaccharide from algae (OSA) on the jejunal disaccharidase activity of piglets (*n* = 6) U/mgprot.

Items		Lactase	Sucrase	Maltase
CON		394.68 ^c^	526.59 ^c^	688.91 ^c^
OSA200		431.25 ^c^	616.18 ^c^	828.88 ^c^
OSA400		534.32 ^b^	870.03 ^b^	924.51 ^bc^
OSA600		691.95 ^a^	1100.70 ^a^	1168.60 ^ab^
OSA800		680.81 ^a^	1285.81 ^a^	1230.22 ^a^
SEM		6.678	19.366	26.868
*p*-Value	Treatment	<0.001	<0.001	0.004
	Linear	0.005	0.048	0.029
	Quadratic	0.022	<0.001	0.003

SEM, Standard error of the means. ^a,b,c^ Different letters mean significantly different (*p* < 0.05).

## Data Availability

Upon reasonable request, the datasets of this study can be available from the corresponding authors.

## Abbreviations

The following abbreviations are used in this manuscript:

OSA	oligosaccharide from algae
ADJ	average daily gain
ADFI	average daily feed intake
F/G	feed-to-gain ratio
CP	crude protein
DM	dry matter
OM	organic matter
EE	ether extract
GE	gross energy
NPU	net protein utilization
BV	biological Value
HDL	high density lipoprotein
LDL	low density lipoprotein
TG	triglyceride
TC	total cholesterol
GLU	glucose
BUN	blood urea nitrogen
MDA	malondialdehyde
GSH-Px	glutathione peroxidase
T-SOD	total superoxide dismutase
IgA	immunoglobulin A
IL-1β	interleukin 1β
IL-6	interleukin 6
IL-10	interleukin 10
TNF-α	tumor necrosis factor α
TFF3	intestinal trefoil factor
HM	histamine
sIgA	secretory immunoglobulin A
TGF-α	transforming growth factor α
LA	lactase
SA	sucrase
MA	maltose enzyme
CTAB	cetyl trimethylammonium bromide
TEAA	total essential amino acids
IMO	isomaltooligosaccharide
